# Androgen-induced alterations in endometrial proteins crucial in recurrent miscarriages

**DOI:** 10.18632/oncotarget.24821

**Published:** 2018-05-15

**Authors:** Tanzil Ur Rahman, Kamran Ullah, Meng-Xi Guo, Hai-Tao Pan, Juan Liu, Jun Ren, Lu-Yang Jin, Yu-Zhong Zhou, Yi Cheng, Jian-Zhong Sheng, He-Feng Huang

**Affiliations:** ^1^ The Key Laboratory of Reproductive Genetics (Zhejiang University), Ministry of Education, Hangzhou, China; ^2^ Department of Pathology and Pathophysiology, School of Medicine, Zhejiang University, Hangzhou, China; ^3^ The International Peace Maternity and Child Health Hospital, School of Medicine, Shanghai Jiao Tong University, Shanghai, China; ^4^ Shaoxing Women and Children's Hospital, Shaoxing, China; ^5^ Department of Zoology, University of Swabi, Anbar, Khyber Pakhtunkhwa, Pakistan

**Keywords:** androgen, recurrent miscarriage, PCOS

## Abstract

High androgen level impairs endometrial receptivity in women experiences the recurrent miscarriage. The mechanism of androgen actions on endometrium is still uncertain. We hypothesized that androgen has a direct effect on the endometrium in women with recurrent miscarriage. In the present study, we assess the impact of androgen (A_2_) at high concentration (10^–7^ M) on Ishikawa cells compared with the physiological concentration of androgen (10^–9^ M). To go into deeper analysis, we use global stable isotopes labeled profiling tactic using iTRAQ reagents, followed by 2D LC-MS/MS. We determine 175 non-redundant proteins, and 18 of these were quantified. The analysis of differentially expressed proteins (DEPs) identified 8 up-regulated proteins and 10 down-regulated in the high androgen group. These DEPs were examined by ingenuity pathway (IPA) analysis and established that these proteins might play vital roles in recurrent miscarriage and endometrium receptivity. In addition, proteins cyclin-dependent kinase inhibitor 2a (CDKN2a), endothelial protein C receptor (EPCR), armadillo repeat for velocardiofacial (ARVCF) were independently confirmed using western blot. Knockdown of *CDKN2a* significantly decreased the expression level of CDKN2a protein in ishikawa cells, and decreased migration (*p* < 0.01), invasion (*p* < 0.05), proliferation (*p* < 0.05), and the rate of Jar spheroid attachment (*p* < 0.05) to Ishikawa cell monolayer. The present results suggest that androgen at high concentration could alter the expression levels of proteins related to endometrium development and embryo implantation, which might be a cause of the impaired endometrial receptivity and miscarriage.

## INTRODUCTION

Polycystic ovarian syndrome (PCOS) is a typical hormonal disorder, touching almost 5%–10% women of reproductive stage [1]. The main features of PCOS comprise irregular menses, oligo/anovulation, and raised circulating androgens. Miscarriage rates have been reported 30–50% of all conceptions in women with PCOS [2, 3]. Data showed that over 30% of women were PCOS who miscarry [4]. Women with high androgens concentrations have higher rates of implantation failure and miscarriage [5].

Research data have shown that androgens were present in the uterine environment during early pregnancy among mammals, including humans, rats, and mice [6–9]. In the mice uterus, quantitative *in situ* hybridization identified uniform labeling of androgen receptor (AR) mRNA in all compartments, including the epithelium [10]. A recent study described nuclear stromal AR staining in the mouse uterus but was not detected in the luminal or glandular epithelium [11]. Female global AR knockout mice are sub-fertile [12]. AR involved in cytoskeletal organization, cell sense, and regulation of cell cycle. AR-dependent signaling is reported to regulate the motile phenotype of decidual cells [13]. In a mouse delayed-implantation model, low androgens may delay embryo attachment, whereas excess androgens lead to an abnormal gene expression in the implantation sites [14].

Recurrent miscarriage (RM) is the loss of three or more successive pregnancies [15]. Observation reporting that conditions related to elevated testosterone, such as PCOS and obesity, have been shown to be associated with higher than predictable miscarriage rates [16]. It is also been declared that there is no evidence of higher aneuploidy rates in the embryos of women with PCOS who miscarried [17] and no increase in rates of miscarriage have been perceived when women with PCOS act as oocyte donors [18]. These results suggest that the effects of raised androgen levels on the endometrial environment may be a cause of miscarriage in women with PCOS [19].

Numerous studies have explored the elevated androgen levels could be observed in women with RM [20, 21]. It has been reported that plasma androstenedione and testosterone concentrations in the women with RM were higher in follicular phase [22]. Okon M.A, *et al* (1996) disclosed that androgens levels in the women with RM were highest during the follicular phase of the cycle [23]. Compare with the total androgen measurements, the testosterone sex hormone binding globulin (SHBG) ratios were enlarged considerably in both the follicular and luteal phases of the cycle. In women, deficiency or excess androgens may contribute to pregnancy- and fertility-related complications such as PCOS [24, 25], endometriosis [26], and recurrent pregnancy loss.

As we know from previous studies that higher androgen levels are lethal in endometrium and cause RM, we conduct an experiment on ishikawa (IK) cells which were treated with A_2_ at 10^–9^ M (a physiological concentration) and 10^–7^ M (high concentration), respectively. We directed a more forceful and accurate method of protein expression, using isobaric tags coupled with 2D LC-MS/MS to screen the relative proteomic profiling of IK cells treated with 10^–9^ M or 10^–7^ M A_2_. The IK cells have the features of glandular and luminal epithelium and apical adhesiveness to Jar cells and serve as an exceptional model for *in vitro* study of endocrine signals in the endometrium [27]. The proteomic results, combined with western blotting and inhibition of certain genes will not only scrutinize the high connection of androgen with endometrium but also should gain new understanding into this complex process of RM.

## RESULTS

### Global profiling of proteins in IK cells

In order to make sure the proteomic changes in the IK cells, we performed iTRAQ analysis to categorize proteomic alterations by comparing the DEPs between the two groups of IK cells treated with 10^–9^ M and 10^–7^ M A_2_, respectively. LC-MS/MS was used to quantitatively spot and map proteins in IK cells (Supplementary Data 1). Using untargeted proteomic analysis, we recognized 175 non-redundant proteins in the IK cells with high confidence (one or more exceptional peptides with false discovery rate (FDR) less than 1%), and 18 proteins were quantified (Table 1).

**Table 1:** Differentially expressed proteins in endometrial epithelial cells identified from iTRAQ analysis

Uni prot accession	Gene symbol	Name	Unique peptides	% coverage	Fold change
**G9BZK0**	*GAPDH*	Glyceraldehyde-3-phosphate dehydrogenase	2	2.55	1.322
**O14578-3**	*CIT*	Citron Rho-interacting kinase	1	1.47	–1.211
**B4DMQ7**	*TP53I3*	Tumor protein p53 inducible protein 3, isoform CRA_c	2	5.89	–1.201
**B7WP27**	*DCPS*	Pre-mRNA-splicing factor CWC22 homolog	1	5.82	1.259
**B7Z889**	*NLRX1*	cDNA FLJ61648, highly similar to Homo sapiens NOD9 protein (NOD9	2	5.64	1.252
**Q13907**	*IDI1*	Isopentenyl-diphosphate Delta-isomerase 1	1	5.19	1.24
**C9JJX6**	*ARVCF*	Armadillo repeat protein deleted in velo-cardio-facial syndrome	1	13.33	1.309
**Q9UNN8**	*PROCR*	Endothelial protein C receptor	1	12.4	–1.251
**B4DPM5**	*SCLY*	cDNA FLJ52960, highly similar to Homo sapiens selenocysteine lyase	1	27.5	1.483
**Q5JR59**	*MTUS2*	Microtubule-associated tumor suppressor candidate 2	2	8.01	–1.819
**H0YD14**	*MYOF*	Myoferlin	2	1.88	–1.312
**G1UI26**	*NBAS*	Neuroblastoma-amplified sequence	1	2.88	1.289
**O43818**	*RRP9*	U3 small nucleolar RNA-interacting protein 2	1	0.99	1.331
**Q92665**	*MRPS31*	28S ribosomal protein S31, mitochondrial	1	1.98	–1.286
**B3KRJ9**	*SREK1*	cDNA FLJ34439 fis, clone HLUNG2001146, highly similar to Splicing factor	1	7.05	1.358
**c9JJX6**	*SPG7*	cDNA FLJ37308 fis, clone BRAMY2016386, highly similar to Paraplegin	2	4.59	–1.22
**Q5ZEY9**	*CDKN2A*	Cyclin-dependent kinase inhibitor 2A	1	7.14	–1.247
**Q5SZE2**	*CERS2*	Ceramide synthase 2	1	4.18	1.261

### Identification of significant DEPs

**Figure 1:**
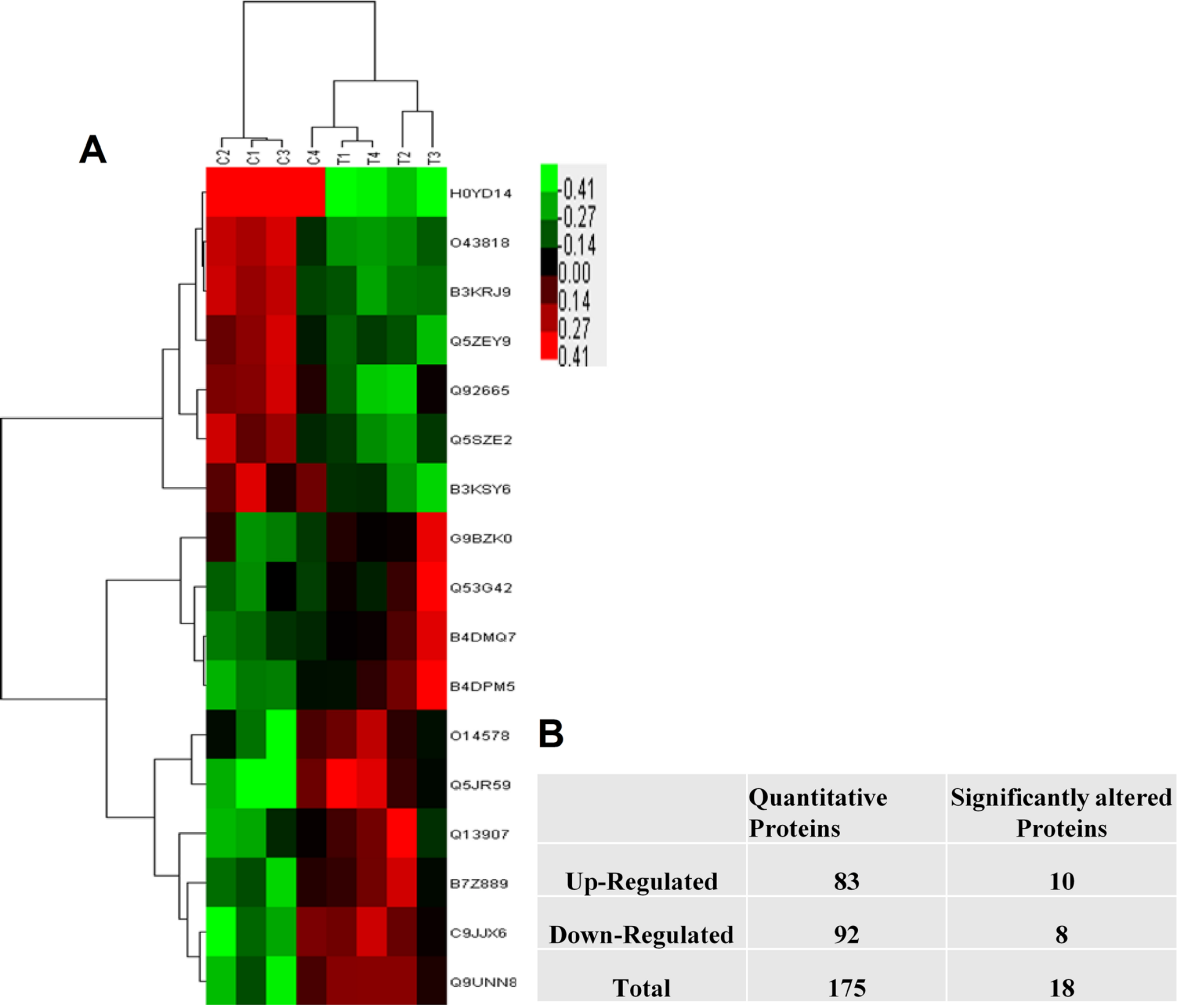
The statistic of the proteins identified in IK cells (**A**) The number of identified proteins. Red show up-regulated proteins while green represents down-regulated proteins. (**B**) Represent the total proteins and Clustering was based on the expression levels of proteins that were analyzed by the feature selection. Bar color represents a logarithmic scale from –3.0 to +3.0.

Using WEKA software, we found 18 significant DEPs. To identify the DEPs between the two different androgen-treated groups, we investigated the expression patterns of 18 proteins. By describing the specific and unique expression patterns of 18 proteins, we were able to group these proteins into two clusters according to their expression patterns (increased expression and decreased expression) (Figure 1B). Among all these proteins, 10 proteins were up-regulated and 8 proteins were down-regulated in IK cells treated with 10^–7^ M A_2_ compared with 10^–9^ M A_2_. The K means clustering of these DEPs, visualized in a heat map (Figure 1A). The acquired heat map (Figure 1A) also displays that the DEPs are from two different treated groups.

### Bioinformatics analysis

**Figure 2:**
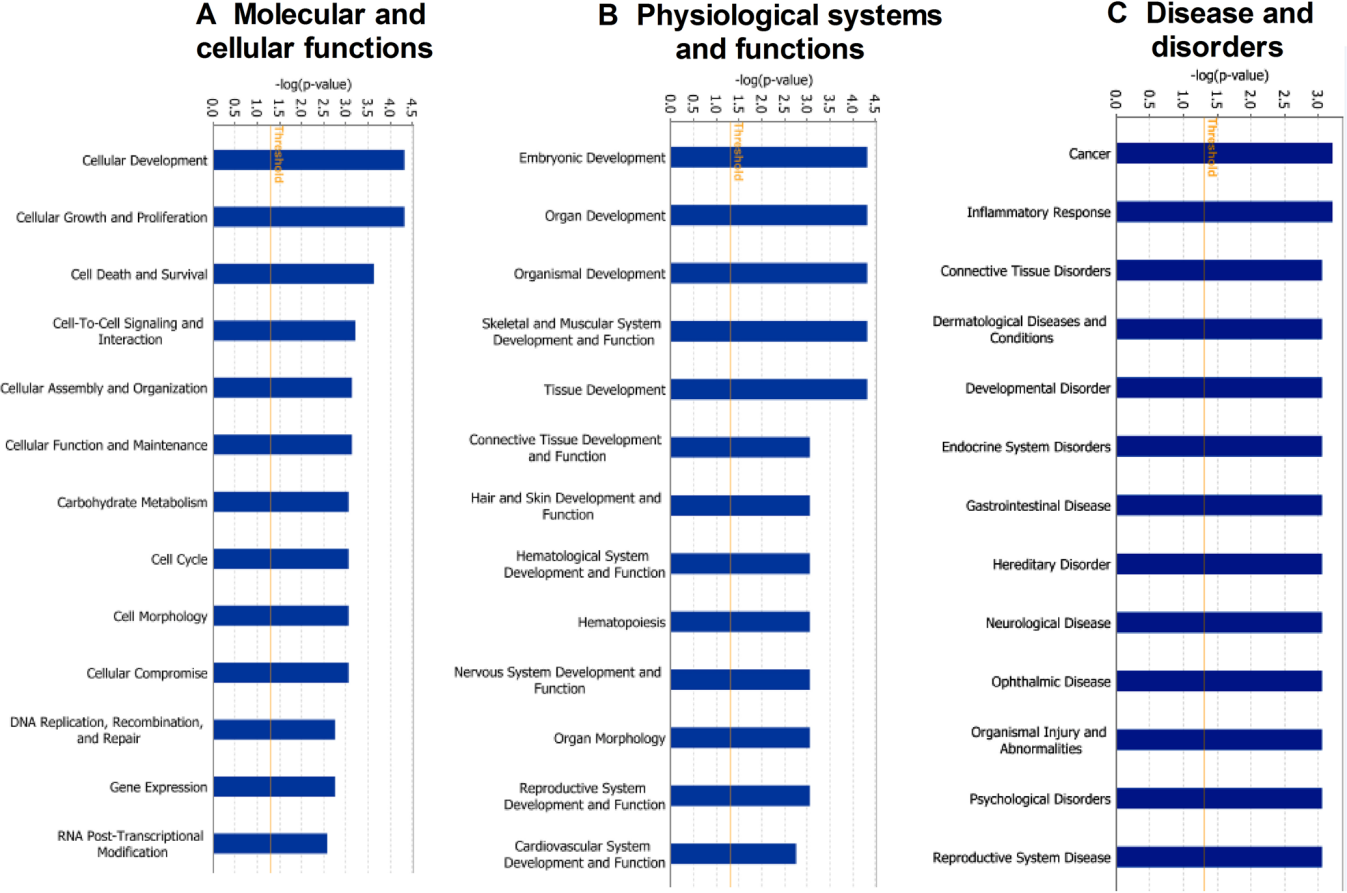
Functional classification of differentially expressed proteins between androgen doses (10^-7^ and 10^-9^ M) to IK cells using ingenuity pathway analysis (**A**) Molecular and Cellular functions (**B**) Physiological System and Functions (**C**) Disease and Disorders.

**Figure 3:**
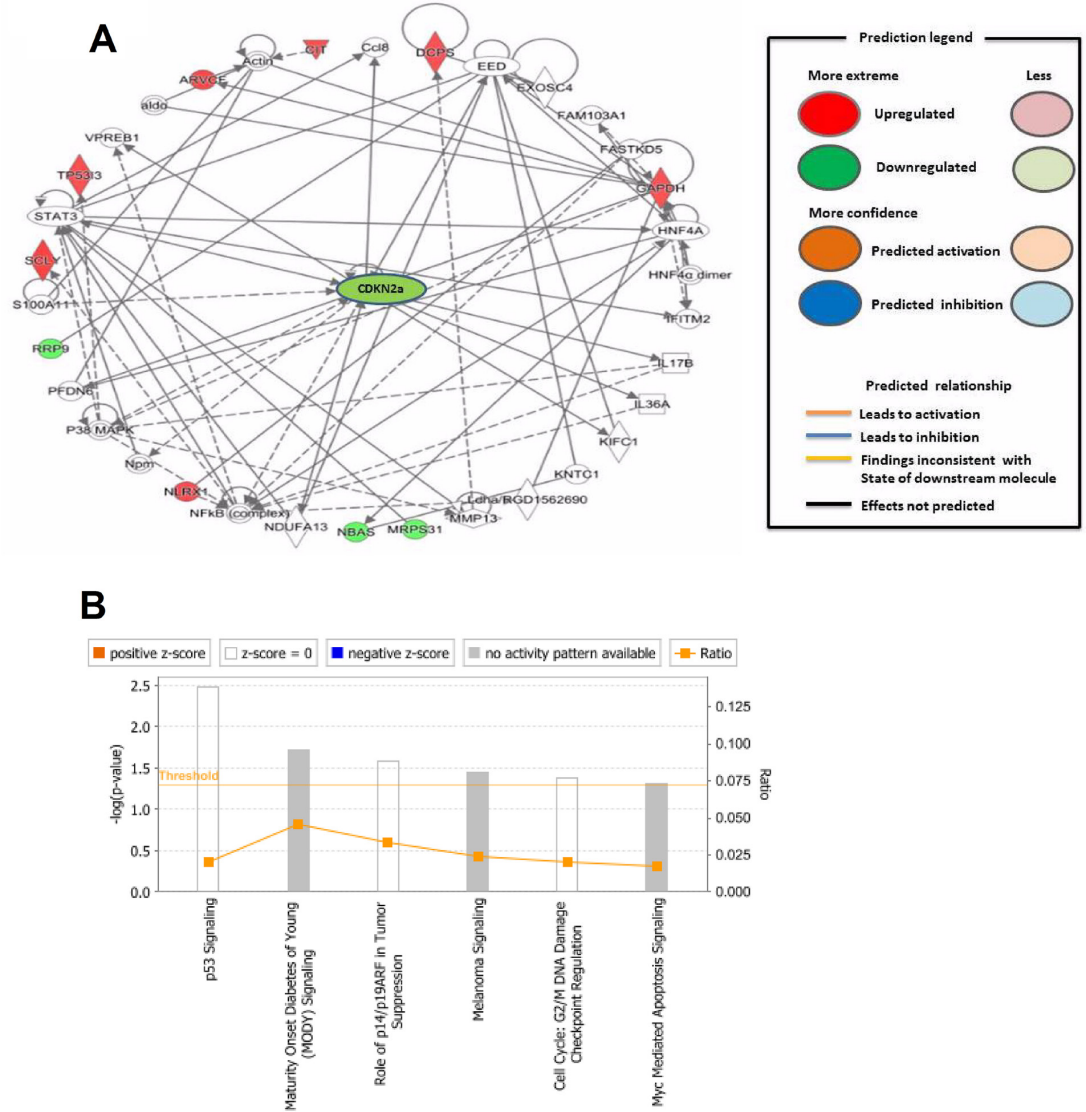
(**A**) Downstream effect analysis of specific differentially expressed proteins associated with recurrent miscarriage and pregnancy complications. The downstream network plays a crucial role in recurrent miscarriage. For this network, genes or gene products are represented as nodes, and the biological relationship between two nodes is represented as an edge. All edges are supported by at least one publication as stored in the ingenuity knowledge database. (**B**) Represent the signaling pathways involved in miscarriage. The intensity of the node color indicates the degree of up- (red) or down- (green) regulation. The legend of the interaction network and the relationships between molecules are summarized on the right of the figure.

For the deeper understanding of these 18 DEPs, we applied IPA. The IPA generated a list of proteins was categorized in “Molecular and Cellular Functions” (Figure 2A), “Physiological System Development and Functions (Figure 2B), and “Disease and Disorder” (Figure 2C). The overlapping *p*-values showed that the DEP associated to 25 subgroups of “Disease and Disorder”, 24 subgroups of “Molecular and Cellular Functions”, and 23 subcategories of “Physiological System Development and Functions” (Figure 2). In the “Physiological System Development and Functions” class, the top three systems included embryonic development, organ development, and organismal development. We also established a network (Figure 3A) and six canonical pathways including p53 signaling, maturity onset diabetes of young signaling, melanoma signaling, p14/p19ARF tumor suppressor signaling, G2/M DNA damage check points and Myc mediated apoptosis signaling (Figure 3B). The bioinformatics analysis suggested that these DEPs from the IK cells might be associated with the RM and endometrial dysfunctions. We found that CDKN2a protein might play important roles in the network (Figure 3A).

### Upstream analysis

The upstream regulator in the IPA may affect other molecules either in direct or indirect ways. Upstream regulators cover a range of molecule types found in the literature, from transcription factors to cytokines, microRNAs, receptors, kinases, chemicals, and drugs. In our study, based on 18 proteins, P38-MAPK and TP53 were the main upstream regulators, and one very important network was the TGF-β1. P38-MAPK regulated CDKN2a while TP53 could activate CDKN2a, glyceraldehyde 3 phosphate dehydrogenase (GAPDH), myoferlin (MYOF), leucine repeat containing X1 protein (NLRX1), and tumor protein p53 inducible protein 3 (TP53I3) (Table 2). All these upstream regulators may be related to RM.

**Table 2:** Up-stream regulator identified from iTRAQ proteomics analysis

Upstream regulator	Molecule type	Predicted activated state	Activation z-score	*P*-value of overlap	Target molecules in dataset
**TP53**	transcription regulator	Activated	1.477	3.48E-03	CDKN2a, GAPDH, MYOF, NLRX1, TP53I3
**P38 MAPK**	group	-	-	2.93E-02	CDKN2a, TP53I3
**CDKN1A**	kinase	-	-	6.11E-04	CDKN2a, CIT, TP53I3
**TGF-β**	kinase	-	-	8.99E-03	CDKN2a, MYOF

### Western blotting validation

**Figure 4:**
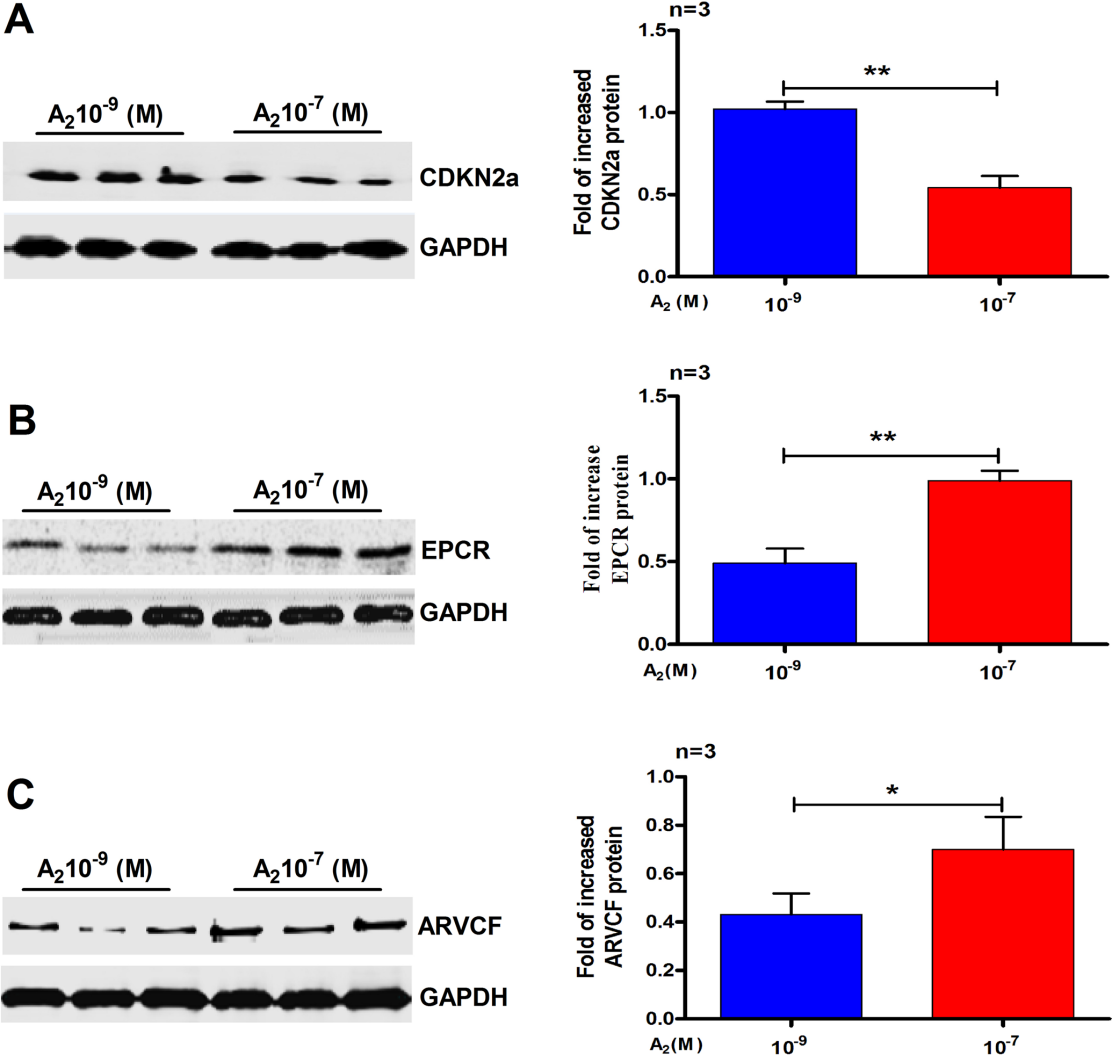
Validation of the differential expression of three selected proteins by Western blots analysis (**A**) CDKN2a (**B**) EPCR and (**C**) ARVCF. Data are present as mean ± SD (*n* = 3). ^*^p < 0.05, ^**^p < 0.01, Student *t*-test.

**Figure 5:**
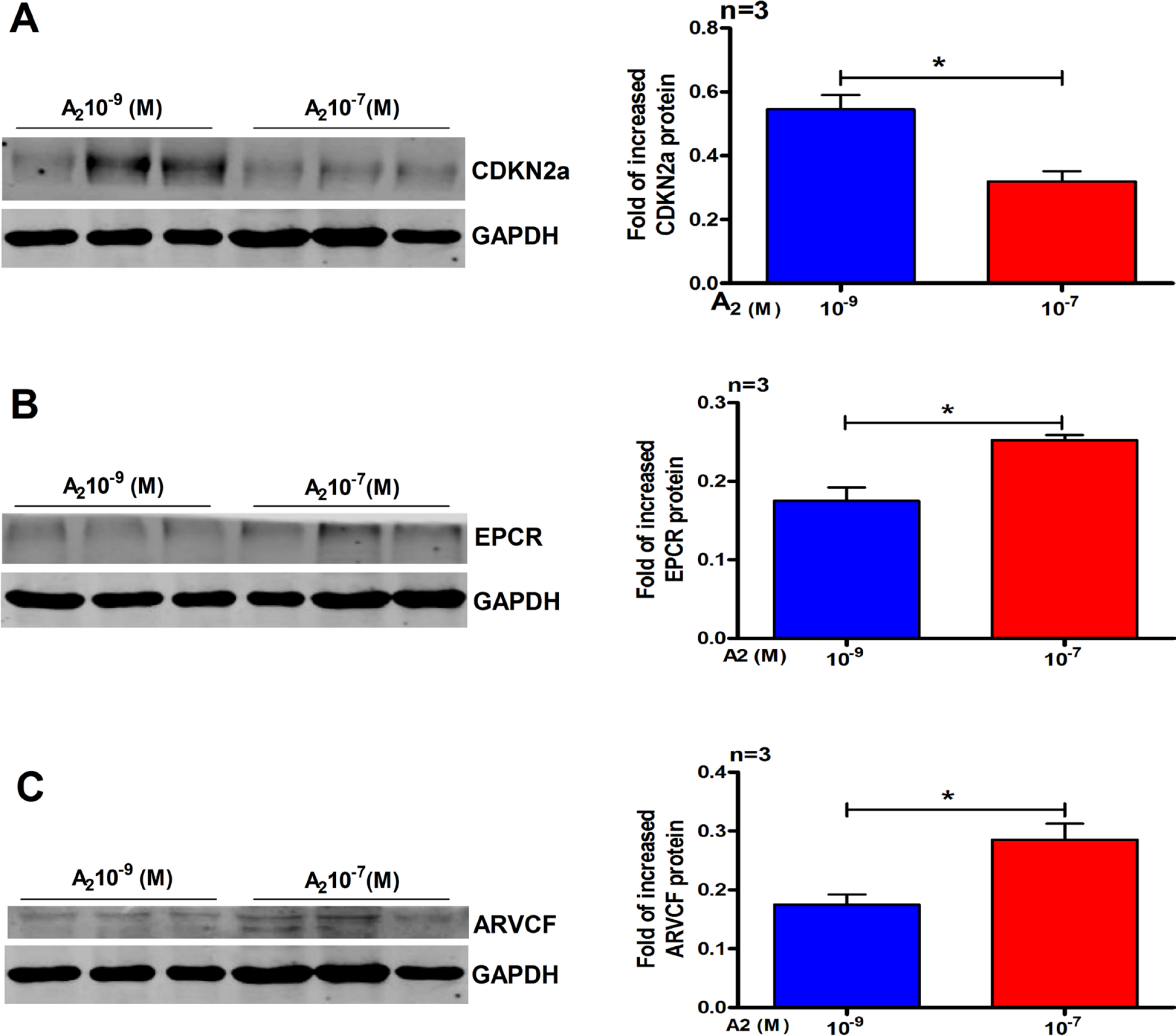
Validation of three selected differentials expressed proteins in primary human endometrial cells by western blot analysis (**A**) CDKN2a, (**B**) EPCR and (**C**) ARVCF. Data are present as mean ± SD (*n* = 3). ^*^p < 0.05, Student *t*-test.

After iTRAQ analysis, we performed western blot to confirm the proteomic differences. As the DEPs were identified from the IK cells, we examined the expression of CDKN2a, endothelial protein C receptor (EPCR) and armadillo repeat protein deleted in velo-cardio-facial syndrome (ARVCF), which have been known to play a very vital role in RM and implantation. As shown in Figure 4, the western blot results were essentially in agreement with iTRAQ results. We also performed the same experiments using primary cultured human endometrial cells. Western blot analysis confirmed that, compared with 10^–9^ M A_2_ treatment, CDKN2a (Figure 5A) was down-regulated by 10^–7^ M A_2_, while EPCR and ARVCF (Figure 5B, 5C) were up-regulated by 10^–7^ M A_2_.

### Protein expression in human endometrial tissue and cells

**Figure 6:**
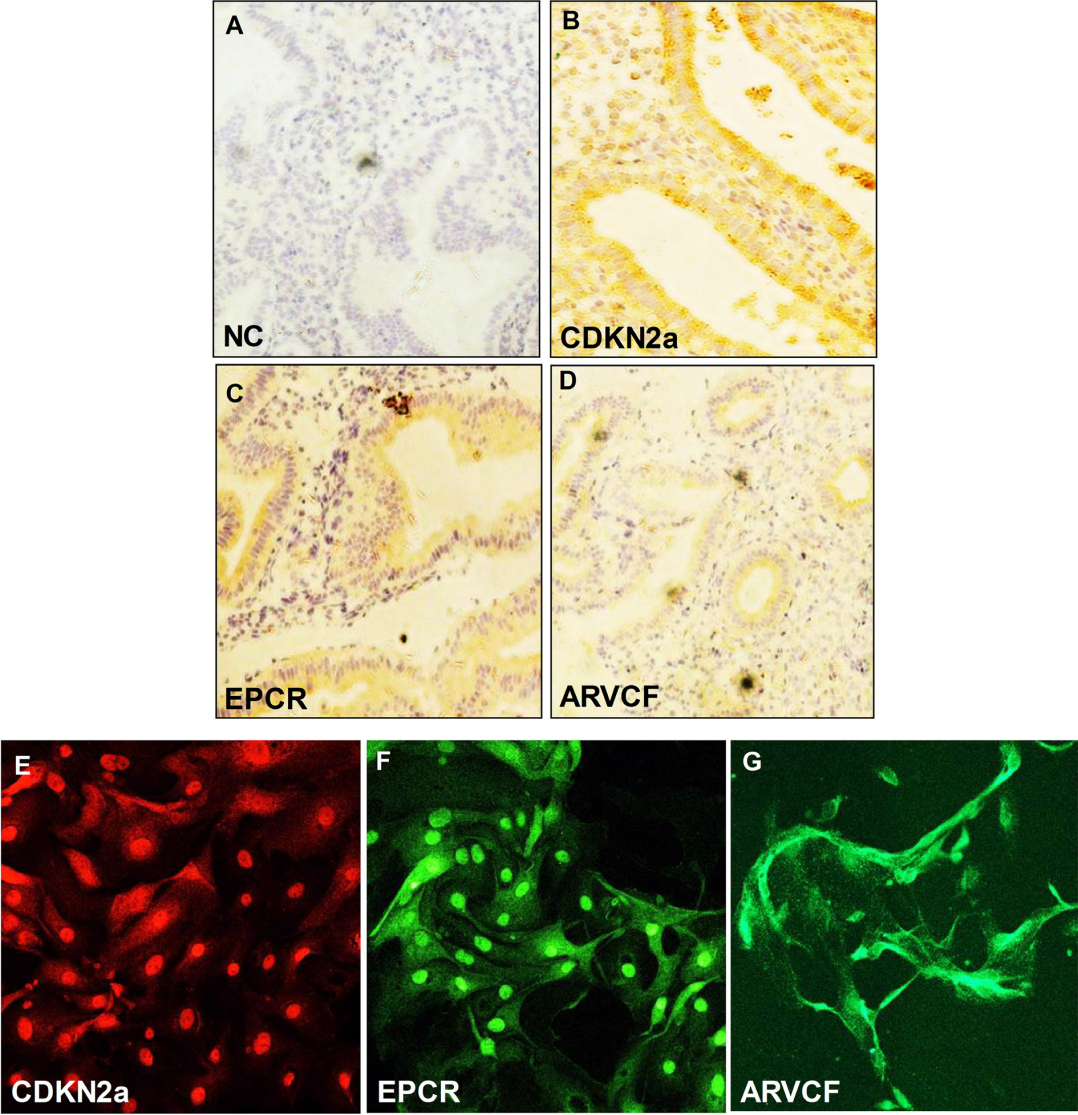
Immunochistochemical analysis of (**A**) CDK2a, (**B**) EPCR and (**C**) ARVCF expression in endometrial epithelial cells. The arrow shows the expression of proteins in the endometrium. (Magnification: ×200). Immunofluorescence analysis of (**D**) CDK2a, (**E**) EPCR and (**F**) ARVCF expression in primary human endometrial cells (Magnification: x100). (**G**) negative control.

We examined the expression pattern of CDKN2a, EPCR, and ARVCF proteins in endometrial epithelial tissues from the donor female. The photomicrographs with immunohistochemical staining showed negative control (Figure 6A), CDKN2a (Figure 6B), EPCR (Figure 6C) and ARVCF (Figure 6D). CDKN2a, EPCR and ARVCF proteins were mainly localized at the endometrial epithelium. We also performed immunofluorescence for the same protein using primary cultured human endometrial cells and found that CDKN2a (Figure 6E), EPCR (Figure 6F) and ARVCF (Figure 6G) were expressed in primary human endometrial cells.

### The effects of knockdown of CDKN2a on cell migration, proliferation, invasion, and Jar spheroid attachment

**Figure 7:**
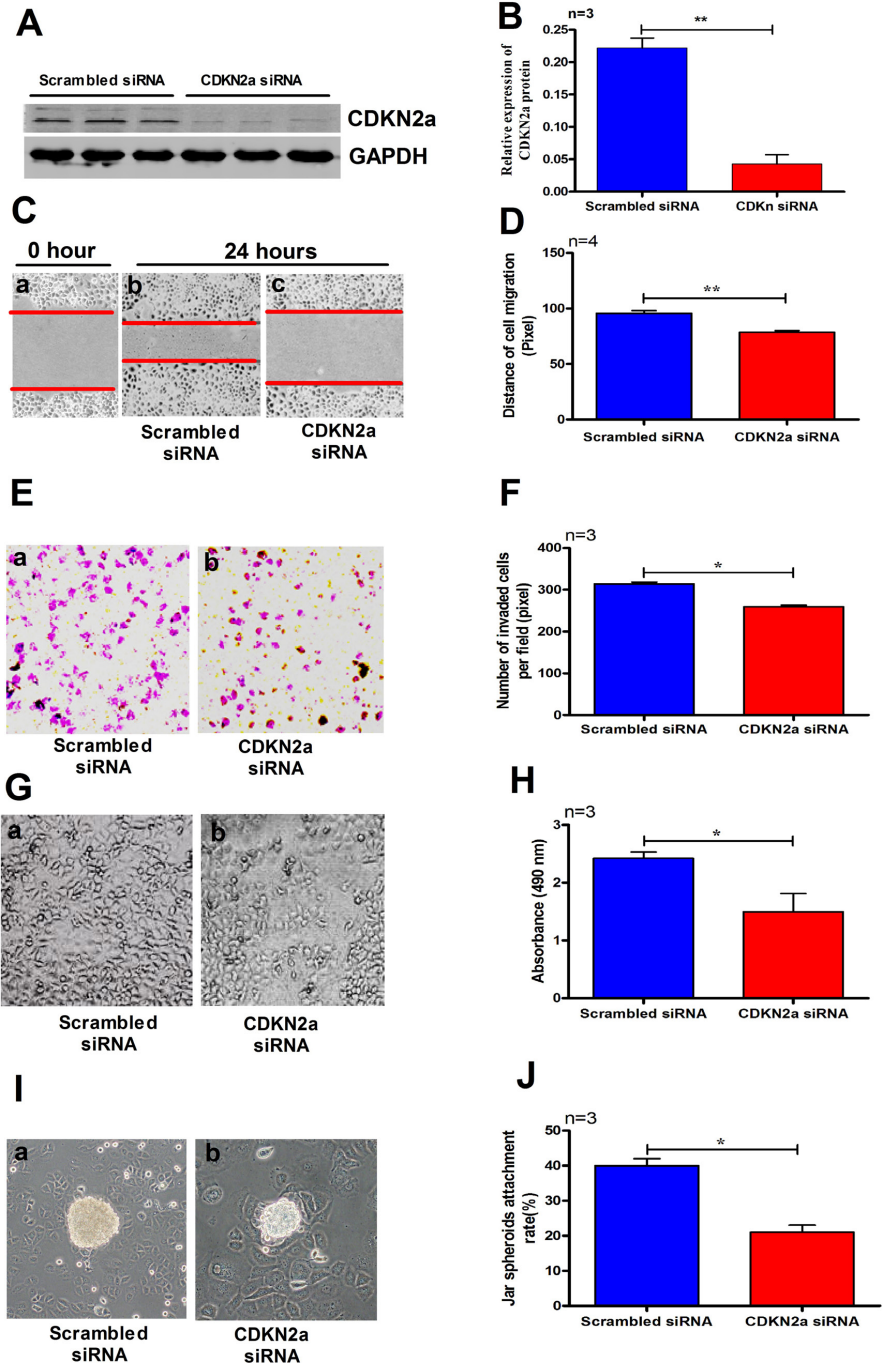
Treating IK cells with *CDKN2a* siRNA significantly reduced the expression level of CDKN2a (**A–B**). A_2_ (10^−7^ M) significantly decreased cell migration (**C–D**), invasion (**E–F**), proliferation (**G–H**) and Jar spheroids attachment (**I–J**) to IK cell monolayer. The data are presented as mean ± SD, ^*^p < 0.05, ^**^p < 0.01, Student *t-*test.

In order to determine the roles of CDKN2a in cell migration, invasion and proliferation of IK cells, we transfected siRNA targeting *CDKN2a* into IK cells. Compared with the cells transfected with scrambled siRNA, treatment of IK cells with *CDKN2a* siRNA for 36 h significantly reduced the expression levels of CDKN2a protein (Figure 7A–7B). To explore the role of CDKN2a in RM, gene-specific siRNA was applied to IK cells in the experiments of cell migration, proliferation, invasion, and Jar spheroid attachment assay. As expected, Knockdown of *CDKN2a* significantly decreased cell migration (Figure 7C–7D, *p* < 0.01), invasion (Figure 7E–7F, *p* < 0.05), proliferation (Figure 7G–7H, *p* < 0.05), and the rate of Jar spheroid attachment to Ishikawa cell monolayer (Figure 7I–7J, *p* < 0.05).

## DISCUSSION

The etiology of RM is poorly understood but may include chromosomal aberrations, uterine abnormalities, thrombosis, and endocrine factors [28]. A relationship between RM and polycystic ovaries has been described by several groups [3]. Data from previous research showed that women with PCOS have a high first trimester RM rate [29]. Hypothetical causes for this high miscarriage rate include elevated testosterone, androstenedione levels, and low level of progesterone [29, 30]. The pathogenesis of RM is very complex and involves the interaction of several genetic and endocrine aspects [31, 32] but their pathogenicity is still largely unknown.

The previous study demonstrated that androgen disrupted endometrial compartments in women who experienced RM [33]. The results from our LC-MS/MS experiment illustrated that the expression levels of many proteins in endometrial cells were androgen-dependent, which might be associated with RM directly or indirectly. We verified the expression of CDKN2a, EPCR and ARVCF in the endometrial epithelial tissues from the donor women. After knocking down the expression of *CDKN2a* we found that the cell migration, invasion, and proliferation of the IK cells were significantly reduced, and the rate of Jar spheroid attachment to IK cell monolayer was significantly decreased.

Several studies showed that cell cycle is progressively regulated by a series of proteins called cyclin-dependent kinase (CDK), whose activities are inhibited by potential inhibitors including CDKN2a/1a [34]. Previous research illustrated that CDK inhibitors played an important role in numerous central cellular routes, including cell proliferation, cell differentiation, and apoptosis [35]. In this study, we found that high concentration of androgen reduced CDKN2a levels in human endometrial cells. Knockdown of *CDKN2a* ultimately reduced migration, proliferation and invasion of IK cells, and decreased the rate of Jar spheroid attachment to the IK cell monolayer. A study has revealed that inhibition of *CDKN2a* in endometrial cells arrested the embryo at 8 cell stage [35]. The results from the present study and other studies suggest that reduced CDKN2a level in endometrium might play an important role in RM of women with high androgen levels.

The recent analysis described that p53 gene influenced cell growth retardation, apoptosis, cell differentiation and DNA repair in RM [36]. It has been detected that mRNA and protein expression levels of p53 and CDKN2a in the RM group were significantly higher than those in the control group [37]. The p53 gene regulates downstream genes including *CDKN1a/2a*, which are important in the apoptosis signaling pathways. Further, it has been clarified that p53 mediates expression of CDK inhibitors, leading to cell arrest in the G1 phase and subsequent cell apoptosis in RM [38]. As in our study, the proteomics results suggest that high androgen level (10^–7^ M) may activate P53 pathway and down-regulate CDKN2a, so the above observation supports our present results. Yang, H et *al.* [39] establish that mouse endometrium expressed *CDKN2a* gene during early pregnancy and it has a possible role in blastocyst implantation [39]. Inclusive mechanisms of CDKN2a in inducing apoptosis of the endometrial cells and contact with the other factors that influence the embryo blastocyst implantation required further research.

Upstream regulators also play a key role in pregnancy complications. Jenny A et al. [40] examined that blockade of p38 MAPK delay mouse implantation process. These results explain downstream targets of p38 MAPK during implantation and indicate that p38 MAPK pathway regulates Trp53 and CDKN2a expression. It has been described that JAK–STAT5 signaling pathway has a key function in preimplantation embryo development, always susceptible to RM [41]. The previous study proved that a TGF-β1 level was increased in maternal plasma during pregnancy, but decrease rapidly later birth [42, 43]. It has been reported that TGF-β1 level was increased in three out of eight nonpregnant women with a past history RM [42]. Other studies have observed that TGF-β1 was expressed in the placenta of RM cases, often with conflicting findings [44]. A study by Magdoud K. *et al* (2013) showed that TGF-β1 was positively correlated with RM [45] but the mechanisms regulating this axis need further investigations.

In conclusion, this is the first study showing the androgen-dependent changes of proteins in endometrial compartments linked to RM. Our findings are consistent with the hypothesis that adverse reproductive outcome in women with hyperandrogenemia experiences RM may be due to a direct detrimental effect of androgens on the endometrium. The proteomic results will also provide a pathway to putative biomarkers for improved RM. However, further molecular and clinical research is needed to elucidate the androgen-dependent mechanism underlying common complex conditions in RM.

## MATERIALS AND METHODS

### Cells culturing

This research study was approved by the research and ethical committee of the Women’s Hospital, Zhejiang University, Hangzhou, China. The primary cultured human endometrial cells were obtained from the Women’s Hospital, School of Medicine, Zhejiang University, and were cultured in DMEM/F12 containing 10% bovine serum albumin. The IK cell line (American Type Culture Collection, Manassas, VA, USA) were obtained from Shanghai Institutes for Biological Science, and maintained in RPMI 1640 medium (Gibco; Thermo Fisher Scientific, Inc., Waltham, MA, USA) containing 10% fetal bovine serum (FBS) and 100 U/ml penicillin and streptomycin antibiotics. When the cells reached to full confluence, the medium was replaced by phenol red-free RPMI 1640 supplemented with 10% charcoal/dextran-treated FBS. For hormonal treatments, A_2_ was added to the culture media to a final concentration of 10^–9^ and 10^–7^ M, respectively, for certain durations according to the experimental purposes.

### Protein extraction

Proteins were extracted from both groups of IK cells and primary human endometrial cells, treated with different androgen concentrations, were performed with cell lysis buffer (RIPA lysis buffer) at 95°C for 5 min and followed by sonication on ice. The crude extracts were incubated at 95°C and cleared by centrifugation at 14000×g for 30 min at 15°C. Thereafter, the supernatant was collected and protein concentration was measured by the BCA protein assay reagent kit (Pierce, Rockford, IL, USA).

### Trypsin digestion of polypeptides and iTRAQ labeling

Protein digestion was performed according to the FASP procedure, as described by J.R. Wisniewski [46]. Four biological replicates were included in the analysis. Briefly, 200 μg of total protein samples were diluted in 30 μL 4% SDS, 100 mM Tris-HCl pH 8.0, and 100 mM dithiothreitol solution and heated at 95°C for 5 min. After each sample was cooled to room temperature and loaded onto an ultrafiltration filter (cutoff 10 kDa, Sartorius, Germany). We added 200 μL UT buffer (8 M Urea and 150 mM Tris-HCl, pH 8.0) to the filter and centrifuged it at 14000×g at 20°C for 30 min. Subsequently, 100 μL of iodoacetamide solution (50 mM iodoacetamide in UT buffer) was added for blocking reduced-cysteines and the samples were further incubated for 20 min in darkness. The filters were centrifuged at 14000×g at 20°C for 20 min and washed (twice) with 100 μL UT buffer at 14000×g for further 20 min. 100 μL dissolution buffer (AB Sciex, Framingham, MA, USA) was added to the filter and it was centrifuged at 14000×g at 20°C for 30 min, the step was repeated twice. Finally, 40 μL of trypsin (Promega, Madison, WI, USA) buffer (2 μg trypsin in 40 μL dissolution buffer) was added and the samples were digested overnight at 37°C. Each filter unit was transferred to a new tube and centrifuged at 14000×g at 20°C for 30 min. The resulting peptides concentrations were estimated by UV light spectral density at OD280 [45]. Then, the peptides mixtures were labeled using the 8-plex iTRAQ reagent according to the manufacturer’s instructions (AB Sciex). Four samples from the control group, treated with 10^–9^ M A_2_, were labeled with mass 114, 115, 116 and 117 isobaric iTRAQ tags, while the other four samples (10^–7^ M A_2_) were labeled with mass 118, 119, 120 and 121 isobaric iTRAQ tags. The labeling solution was incubated at room temperature for 2 h before further analysis.

### Strong cationic-exchange chromatography separation

The combined sample was acidified with 1% trifluoroacetic acid before being subjected to strong cationic-exchange chromatography (SCX) fractionation using a PolySULFOETHYL column (4.6 × 100 mm, 5 μm, 200 Å, Poly LC Inc., Columbia, MD, USA). Solvent A consisted of 10 mM KH_2_PO_4_ in 25% (v/v) ACN and solvent B was solvent A with 500 mM KCl added. The solvents were applied using a gradient of 0%–10% solvent B for 2 min, 10–20% solvent B for 25 min, 20%–45% solvent B for 5 min, and 50%–100% solvent B for 5 min. The elution was monitored by absorbance at 214 nm and fractions were collected every 1 min. Finally, these samples were combined into 10 fractions based on the quantity of peptide and then desalted on C18 cartridges (Sigma, Steinheim, Germany). Each SCX salt step fraction was dried in a vacuum centrifuge and reconstituted with 40 μL 0.1% (v/v) trifluoroacetic acid.

### LC-MS/MS analysis

Peptide mixture (5 μg) from each fraction was subjected to nano LC-MS/MS analysis. The mixtures were loaded onto the Thermo EASY-nLC column (Thermo Finnigan, San Jose, CA, USA) (100 mm × 75 μm, 3 μm) in solvent C (0.1% formic acid) and separated with a linear gradient of solvent D (80% acetonitrile with 0.1% (v/v) formic acid) at a flow rate of 300 nl/min over 120 min: 0–100 min with 0% to 45% solvent D; 100–108 min with 45% to 100% solvent D; 108–120 min with 100% solvent D. The Q-Exactive (Thermo Finnigan) mass spectrometer acquired data in the positive ion mode with a selected mass range of 300–800 mass/charge (m/z). Dynamic exclusion was used with 40.0 s duration. Q-Exactive survey scans were set as 70,000 at m/z 200 and 17,500 at m/z 200 of resolution for HCD spectra. MS/MS data were acquired using a data-dependent acquisition method with the top 10 most abundant precursor ions. The normalized collision energy was 30 eV and the underfill ratio was defined as 0.1% on the Q-Exactive.

### Protein identification and quantification

Protein identifications were performed using the MASCOT search engine (version 2.2.1; Matrix Science, London, UK) embedded into Proteome Discoverer 1.3 (Thermo Electron, San Jose, CA, USA), searching against the Uniport database of human protein sequences (03–2013, 133549 entries, downloaded from: http://www.uniprot.org/) and the decoy database. Search parameters were set as follows: monoisotopic mass, peptide mass tolerance at ± 20 ppm and fragment mass tolerance at 0.1 Da, using trypsin as the enzyme and allowing up to two missed cleavages. Variable modifications were defined as oxidation of methionine and iTRAQ 8-plex labeled tyrosine, while lysine and N-term of peptides labeled by iTRAQ 8-plex and carbamidomethylation on cysteine were specified as fixed modifications. FDR of both proteins and peptides identified as set to be less than 0.01. Protein identification was supported by at least one unique peptide.

### Bioinformatics analysis

All DEPs (*p-*value < 0.05) were selected and the ones with the differential expression ratio of over ± 1.2 were retained. The capability of the resulting DEPs in differentiating two groups of samples was then evaluated by hierarchical cluster analysis. For this purpose, the Cluster 3.0 (http://bonsai.hgc.jp/∼mdehoon/software/cluster/software.htm) and the Java Tree view software (http://jtreeview.sourceforge.net) were used. Disease analysis, pathway and network generation were performed using IPA software package (QIAGEN, Redwood 185 City, CA, USA). IPA is a knowledge database relying on published literature related to protein function, localization, relevant interactions and biological mechanisms. Calculated the Z-score can infer the activation states (“activated” or “inhibited”) of implicated biological processes. Fisher’s exact test was used to calculate a *p*-value to determine the probability that the association between proteins in the dataset, and, the biological process could be explained by chance alone.

### Western blot analysis

For western blot analysis, proteins obtain from the IK and primary cultured human endometrium cells were electrophoresed at 20 μg/lane and separated on a 15% SDS gel. After running on a gel, the proteins were transferred to a nitrocellulose transfer membrane (Bio-Rad, Hercules, CA, USA). To protect from non-specific binding the membrane was incubated for 1 h with 5% skimmed milk (Difco) in Tween20 1x TBS (TBST). Next, the membrane was incubated overnight at 4°C with primary antibodies against CDKN2a (Abcam ab108349, Cambridge, UK, 1:1000), EPCR (Abcam, ab174234, Cambridge, UK, 1:2000), ARVCF (Santa Cruz Biotechnology, INC sc-23874, California, USA; 1:1000) and GAPDH (CW bio CW0266A, Beijing, China, 1:1000) at 4°C overnight. After three washes with 1x TBST (pH 7.4), each membrane was incubated with the appropriate secondary antibody (1: 10000) at room temperature for 1 h. After additional three washes, protein intensities were determined and analyzed using Odyssey^®^ Imager (LI-COR, Lincoln, NE, USA).

### Tissue immunohistochemistry (IHC) and cell immunofluorescence analyses

Human endometrial tissues and primary endometrial cells were obtained from donors women at receptive phase. Written informed consent was obtained from all subjects prior to tissue collection, and ethical approval was granted by the Committee of School of Medicine, Zhejiang University. Samples were fixed in 10% formalin and processed for paraffin embedding. Cross-sections (5 µm thickness) were mounted onto microscope slides (Fisher Scientific). After deparaffinization and rehydration, sections were washed three times with phosphate-buffered saline (PBS) for 5 min. Immunohistochemistry was performed on endometrial sections using the LSAB Peroxidase Kit (DAKO, CA, USA). After blocking with 5% bovine serum albumin (BSA), the sections were incubated with the following primary antibody diluted in blocking solution (0.25% BSA, 0.3% Triton X-100, sterile PBS) overnight at 4°C. The primary antibodies included CDKN2a antibody (1:1000), EPCR antibody (1:500) and ARVCF antibody (1:1000). Tissue sections were then washed with PBS for 5 min followed by their respective secondary antibodies with nuclear counterstaining, performed with 4,6-diamidino-2-phenylindole (DAPI, Molecular Probes/Life Technologies, Vienna, Austria). While the cells were grown over coverslip, cells were fixed and incubated with their respective primary antibodies. DAPi was used as chromogens, and photographs were taken by Carl Zeiss-800 microscope (Germany). Assessment of the tissue sections was performed using Leica Light Microscope (Leica Microsystems B353, Optika, Italy).

### Interfering RNAs (siRNAs) knockdown studies

IK cells were seeded in 6-well plates. For knockdown experiments, siRNA targeting the *CDKN2a* (sc-37622) gene (100 pmol/well) and siRNA negative control (scrambled, sc-37007) were purchased from Santa Cruz Biotechnology. Cell transfection was conducted using transfection reagent (sc-29528) and transfection media (sc-36868) (Santa Cruz Biotech, INC) according to the manufacturer’s instructions.

### Wound healing assay

IK cells (1 × 10^5^/well) were seeded in 12-well plates pre-coated with 0.5% gelatin overnight at 4°C. After pretreatment (knockdown of *CDKN2a*), cells were cultured to confluence overnight. The monolayer cells were then scratched with a standard 200 μl pipette tip and washed twice with PBS to remove detached cells. After scratching the lines, cells were cultured for 24 h. Mitocycin C (10 mg/ml) was included in the medium to prevent cell proliferation. Wound healing was quantified by measuring the migratory distance of cells.

### Transwell invasion assay

A permeable filter of transwell system (Corning Incorporated, Midland, MI, USA) was used to study the invasion ability of cells. The inside compartment of the transwell inserts was coated with Matrigel (BD Biosciences, Bedford, MA, USA) at 4°C overnight, and then blocked by 1% BSA/PBS solution for 30 min at room temperature. After pretreatment (knockdown of *CDKN2a*), IK cells (1 × 10^5^/well) were loaded in the upper chamber in culture medium with 0.2% BSA. Cell migration to the other side of the membrane was induced by 30% FBS-containing medium in the lower chamber for 24 h. Cells were fixed in methanol for 30 min and stained with 0.5% crystal violet for 15 min. After gently removing the cells on the upside of the top chamber, migrated cells were photographed and counted with Image-J software (National Institutes of Health, Bethesda, MD, USA).

### Cell proliferation assay

IK cells (1 × 10^5^/well) were plated in 96-well plates. After pretreatment (knockdown of *CDKN2a*), cells were cultured for 24 h in culture medium. The MTT assay was applied to quantify cell proliferation, and the absorbance of samples was measured at 490 nm.

### Attachment assay of jar spheroid to ishikawa cells

*In vitro* attachment model of multicellular spheroids of human choriocarcinoma Jar cells (American Type Culture Collection, Manassas, VA; HTB 144) were applied to IK cells monolayer. Jar cells at 40% confluence in 10 cm plate were transfected with *CDKN2a* siRNAs or scrambled siRNA for 24 h and then made into Jar spheroids according to a standard procedure [46]. Total 100 Jar spheroids were transferred onto the surface of a confluent cell monolayer for 1 h. Non-adherent spheroids were detached by centrifugation (10×g; 10 min) of the six-well plates with the cell surface facing down. We counted the attached spheroids under a light microscope and the results were expressed as the percentage of the total number of spheroids used.

### Statistical analysis

Statistical analysis was carried out using Graph Pad Prism version 5 (San Diego, CA, USA). Statistical significance for comparison between groups was determined by using Student’s *t*-test. All samples were tested in triplicate, and the data are expressed as mean ± SEM. *p* < 0.05 was considered significant.

## SUPPLEMENTARY MATERIALS





## Abbreviations

RM: Recurrent miscarriage
PCOS: Polycystic ovary syndrome
M: Molarity
10^–7^ M or 27.24 pg/ml: picograms per milliliter
10^–9^ M or 0.2724 pg/ml: picograms per milliliter
IK: Ishikawa
FDR: False discovery rate
IPA: Ingenuity pathway analysis
SCX: strong cationic-exchange chromatography
DEPs: Differentially expressed proteins
AR: Androgen receptor
LH: Luteinizing hormone
SHBG: Sex hormone binding globulin
FBS: Fetal bovine serum
TM: Thrombomodulin
