# Supplementary material for: Androgen-induced alterations in endometrial proteins crucial in recurrent miscarriages
Source: Oncotarget. 2018 May 15;9(37):24627–41. doi: 10.18632/oncotarget.24821 (PMC5973874; doi:10.18632/oncotarget.24821)
Supplement: Supplementary file 1 [file oncotarget-09-24627-s001.pdf]

Androgen-induced alterations in endometrial proteins crucial in recurrent miscarriages

SUPPLEMENTARY MATERIALS

Supplementary Data 1: Mascot database search Amino acid reference data. See Supplementary_Data_1
